# The Combined Effects of Amino Acid Substitutions and Indels on the Evolution of Structure within Protein Families

**DOI:** 10.1371/journal.pone.0014316

**Published:** 2010-12-13

**Authors:** Zheng Zhang, Yuxiao Wang, Lushan Wang, Peiji Gao

**Affiliations:** 1 State Key Laboratory of Microbial Technology, Shandong University, Jinan, Shandong, China; 2 Division of Basic Science, UT Southwestern, Dallas, Texas, United States of America; American Museum of Natural History, United States of America

## Abstract

**Background:**

In the process of protein evolution, sequence variations within protein families can cause changes in protein structures and functions. However, structures tend to be more conserved than sequences and functions. This leads to an intriguing question: what is the evolutionary mechanism by which sequence variations produce structural changes? To investigate this question, we focused on the most common types of sequence variations: amino acid substitutions and insertions/deletions (indels). Here their combined effects on protein structure evolution within protein families are studied.

**Results:**

Sequence-structure correlation analysis on 75 homologous structure families (from SCOP) that contain 20 or more non-redundant structures shows that in most of these families there is, statistically, a bilinear correlation between the amount of substitutions and indels versus the degree of structure variations. Bilinear regression of percent sequence non-identity (PNI) and standardized number of gaps (SNG) versus RMSD was performed. The coefficients from the regression analysis could be used to estimate the structure changes caused by each unit of substitution (structural substitution sensitivity, SSS) and by each unit of indel (structural indel sensitivity, SIDS). An analysis on 52 families with high bilinear fitting multiple correlation coefficients and statistically significant regression coefficients showed that SSS is mainly constrained by disulfide bonds, which almost have no effects on SIDS.

**Conclusions:**

Structural changes in homologous protein families could be rationally explained by a bilinear model combining amino acid substitutions and indels. These results may further improve our understanding of the evolutionary mechanisms of protein structures.

## Introduction

The tertiary structure of a protein is determined by its primary sequence, and a certain protein sequence will fold into a unique structure. In the evolution of homologous proteins, variations in protein sequences cause changes in protein structures and functions. Nevertheless, structures are more conserved than sequences and functions. For example, the topology of proteins may not alter significantly even if the sequences differ by 70% [1], [2]. It is widely accepted that there are around 1000 different kinds of protein folds that cover about 10000 different protein sequence families [3], [4], [5]. On the other hand, homologous proteins with similar topology can have different functions [6]. This lead to an intriguing question: how do sequence changes in a homologous family cause variations in structures?

An early study by Chothia & Lesk indicated that the extent of the structural changes observed between two proteins is directly related to the extent of the sequence changes. They proposed that the RMSD of the positions of the α-carbon atoms of the two proteins is exponentially related to the fraction of mutated residues [7]. Later research based on larger dataset yielded similar results [8], [9], [10], [11]. However, Wood & Pearson's work on 36 SCOP structure families revealed a linear relationship between protein sequence similarity and structure similarity by using statistical Z-scores rather than simple measures such as RMSD and percent sequence identity [12]. To address the problem of “fold recognition”, Koehl and Levitt investigated inverse correlations between structure similarity and sequence similarity of 12 protein structure families. They defined the structure similarity between two proteins as the mean distance between the sequences in the subsets of sequence space compatible with their structures, and found that structure variation within a protein family is linearly related to sequence similarity [13]. Recently, Panchenko et al. studied 81 homologous protein families from the Conserved Domain Database and showed that for conserved structural domains, structure changes are linearly related to sequence variations [14]. They also pointed out that for most protein families the loop structure similarity is significantly linearly related to sequence identity [15].

Mutations in DNA sequences include not only base substitutions (transitions and transvertions), but also indels of one or several bases [16]. Indels also play an important role in the process of molecular evolution [17]. Grishin's analysis on evolutionarily related proteins with large structure differences suggested that mechanisms such as insertions/deletions/substitutions, circular permutations, strand invasions/withdrawals, and hairpin flips/swaps emerge as leading causes for structural divergence within homologous families [18]. Our previous study showed that indels can cause structural shift in the flanking regions, with a first-order exponential decay relation between the extent of structural shift and the distance to indels [19].

In this paper, substitutions and indels are treated as two independent factors that cause structure changes, and their combined effects on structure evolution within homologous protein families were studied. We employed the structure alignment program SSM [20] and carried out researches on 75 protein families from the SCOP 1.73 structural classification database [21], [22] (Table S1). All the 75 families have 20 or more native protein structures determined by X-ray crystallography, which belong to the ASTRAL95 non-redundant structure database [23]. Plots of RMSD versus percent sequence non-identity (PNI) and standardized number of gaps (SNG) indicated that in most families both amino acid substitutions and indels have a linear influence on structure variations. The regression coefficients from bilinear regression of PNI and SNG versus RMSD represent the global influence on structure arising respectively from one substitution and one indel in a sequence with standard length. Thus we termed them as structural substitution sensitivity and structural indel sensitivity, respectively. In those families with a high bilinear correlation coefficient (R>0.75) and statistically significant regression coefficients (p<0.01), we studied the factors that cause the regression coefficients to be different between families.

## Results

### Sequence similarity and structure similarity

In the process of genomic evolution, substitutions and indels are two different types of sequence variation, and the frequency of indels is one magnitude lower than that of substitutions [24], [25], [26]. In the evolution of protein structures, both substitutions and indels contribute to structure changes, especially the latter, which may cause significant or even drastic alterations in structures [18]. In our study, we defined the structure similarity between two proteins as the RMSD of Cα in the aligned region. Sequence change is defined as two variables (PNI and SNG) in order to represent the combined effects of substitutions and indels. Both of them are obtained from homologous protein alignments. PNI refers to the ratio of substituted sites in aligned regions, while SNG corresponds to the number of indels that exist in a sequence with standard length (see Methods). SNG is obtained by standardizing the sum of gap numbers in the alignment results, where gap number refers to the number of unaligned regions but not the number of unaligned sites.

Meanwhile, in order to weaken the influence from non-sequence factors, non-redundant structures from the ASTRAL95 database were chosen in our study. In addition, structures determined by NMR and mutated structures were excluded. Also, we focused on high-resolution structures (solved by X-ray crystallography to better than 2.2 Å resolution) for reference.

To reduce the number of false indels arising from wrong alignments, we employed sequence alignment programs based on structures to perform pairwise alignments of non-redundant structures in each family. RMSD, PNI and SNG are all obtained from the alignment results. Gaps produced by sequence alignment programs based on sequences are mainly governed by gap penalty [27], which is not an accurate description of the real gaps appearing in the process of sequence divergence [24]. Sequence alignment programs usually randomly yield a relatively high sequence identity in remote sequence or short sequence alignments, i.e. the “high-order effect” [28]. Because structures are more conserved than sequences, the sequence alignment acquired by matching structures of remotely homologous proteins tends to be more reliable [29].

P-score, a parameter which reflects the statistical significance of alignment results, is reported by the SSM structure-matching program based on the RMSD, the length of the aligned region and the number of gaps. Non-trivial matches are expected to have a P-score greater than 3 [20]. So in our study, we focused on the alignment results with P-score>3 (referred to as the accurate alignments hereafter). We also studied the whole alignment results for comparison.

**Figure 1:**
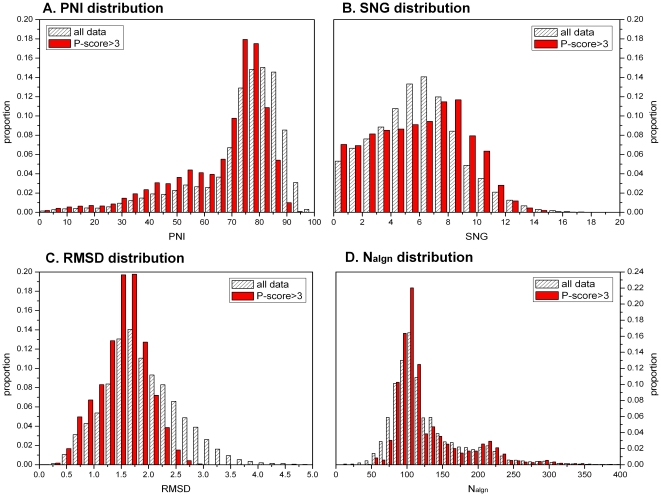
The distribution of PNI, SNG, RMSD and alignment length (N_algn_). A. The distribution of percentage sequence non-identity (PNI). It shows that most of the PNI are relatively high and they range widely. B. The distribution of standardized number of gaps (SNG). Almost all of the alignments we studies have gaps. C. The distribution of RMSD. The structural differences of homologous proteins are relatively very small. D. The distribution of alignment length. We employed accurate alignment, which excludes those alignments with short alignment length. The results are obtained from pairwise alignments of non-redundant structures in 75 families, belonging to “ASTRAL 95 select” database. Filled columns show the 50359 accurate alignments with P-score>3. Shaded columns show the whole 81859 alignments.

We carried out pairwise alignment for non-redundant structures from 75 SCOP families. 81859 alignment results were obtained from the 3179 submitted structures. The distribution of the whole sequence similarity and structure similarity is illustrated in Figure 1. About 28.1% of the accurate alignments (P-score>3) have a PNI≤60%, while in all alignment results, alignments with PNI≤60% account for only 18.2% (Figure 1A). Indels as well as substitutions are observed widely. In all the accurate alignment results there are only 2.4% alignments with no gaps (Figure 1B). SNG increases slowly in the range of 0–9, while for 9–15 it decreases sharply. Although there are a large amout of substitutions and indels, structure changes do not exceed 3 Å in the accurate alignment (Figure 1C). In addition, the alignments with length less than 50 residues are excluded by choosing accurate alignment results (Figure 1D).

In summary, each SCOP family contains a variety of entries ranging from highly similar structures to remotely related homologs. There are many indels among remote proteins which are suitable for the study of the combined effects of sequence substitutions and indels on the evolution of structures.

### Bilinear correlation of sequence variations and structure changes

**Figure 2:**
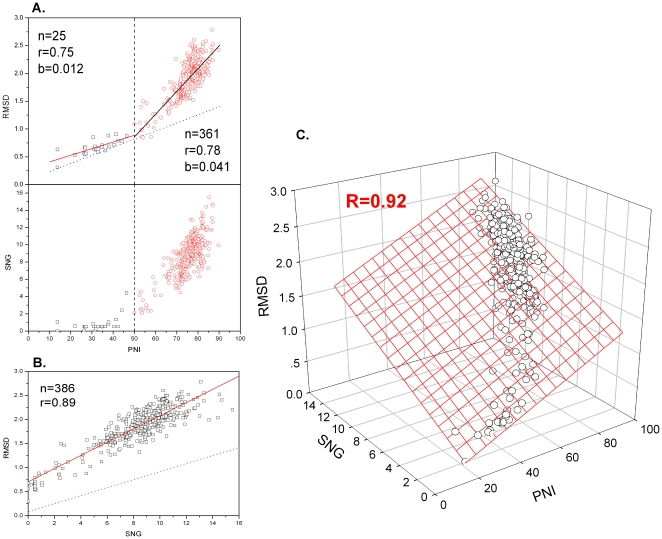
Bilinear correlation of PNI, SNG versus RMSD within the amylase catalytic domain family (AMC). A. The piecewise linear correlation of PNI-RMSD, which is related to the distribution of SNG. B. The linear correlation of SNG-RMSD. C. The bilinear correlation of PNI, SNG versus RMSD, illustrated by 386 accurate alignment results. The dotted lines in A, B show the real influence of PNI and SNG on RMSD, obtained by conducting bilinear regression, respectively.

As shown in Figure 2, the amylase catalytic domain family (AMC, c.1.8.1) is chosen as an example to demonstrate the correlation of PNI, SNG versus RMSD. By considering the correlation of PNI-RMSD separately, we got a piecewise linear correlation but not a fully linear one. The slope is small (b = 0.012) with PNI<50%, but significantly increased (b = 0.041) when PNI is above 50%. In fact, the piecewise linear correlation of PNI-RMSD is closely related to the corresponding distribution of SNG. There are almost no indels when PNI is below 50%. However, indels increase together with substitution when PNI is above 50%. In addition, SNG-RMSD has a significant linear correlation (r = 0.89, p<0.01). If PNI and SNG are treated as two variables, a significant bilinear correlation of PNI, SNG versus RMSD (R = 0.92) is observed in AMC families by conducting bilinear regression.

**Figure 3:**
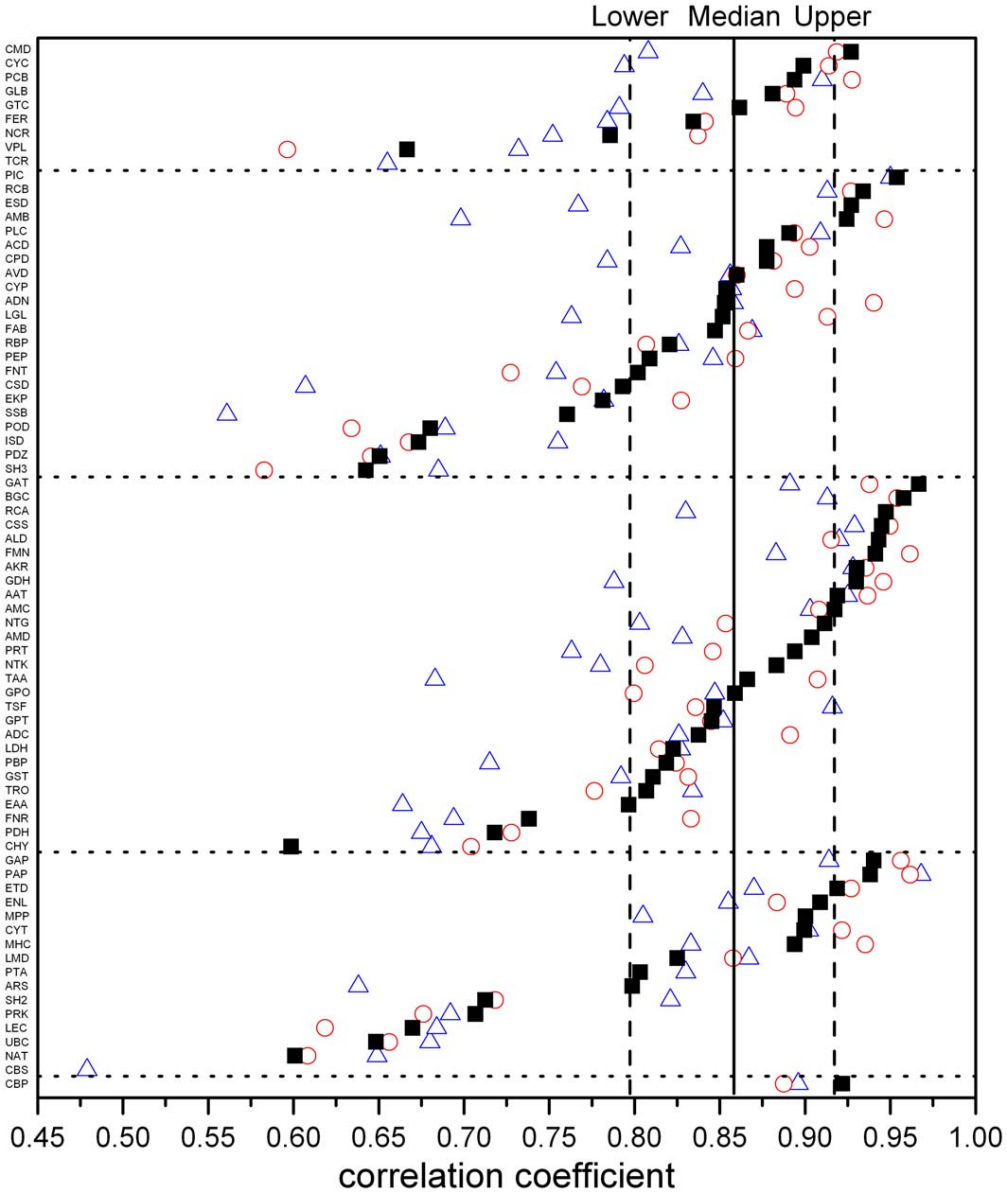
Substitutions and indels are closely related to structural changes. Filled squares show the bilinear multiple correlation coefficients obtained from reasonable structure alignments of families from the “ASTRAL95 select” database. The vertical thick line shows the median bilinear correlation coefficient of 73 families (0.859); the right and left dotted lines show the upper quartile (0.918) and lower quartile (0.798), respectively. Triangles show the bilinear multiple correlation coefficients obtained from all the alignments within each family (75 families). Open circles show the bilinear multiple correlation coefficients obtained from accurate alignments of high-resolution structures (64 families). The five regions from the top to bottom are five SCOP structure classes, which are all α, all β, α/β, α+β and α&β. Abbreviated family names are shown in Table S1.

Table S1 shows the calculated statistical parameters for substitutions, indels and structure changes for all the selected families. Bilinear correlation analysis of PNI, SNG versus RMSD based on the accurate alignment results (P-score>3) shows that in 73 families (97% of the total) the bilinear correlation coefficients are significant (p<0.01). Half of the 73 families have bilinear correlation coefficients greater than 0.859, which means that 74% of the structure changes in those families can be explained by the bilinear model (Figure 3). Similar results were obtained from analysis based on the high-resolution structures available for 64 families (Table 1). Analysis of all the alignment results reveals that in all 75 families the bilinear correlation coefficients are statistically significant (p<0.01), though the median bilinear correlation coefficient is a little lower. This high sequence-structure correlation indicates that in these protein structure families the structure changes are mainly resulted from sequence variations. Moreover, in 63 families (84% of the total) both the PNI-RMSD partial correlation coefficients and SNG-RMSD partial correlation coefficients are significantly different from zero (p<0.01). This indicates that in most families both substitutions and indels cause structure changes.

**Table 1:** The bilinear correlation coefficients of sequence elements and structure.

	Number of families	Median	Upper quartile	Lower quartile
ASTRAL95 select	75 (100%)	0.821	0.869	0.715
ASTRAL95 select & P-score>3	73 (97%)	0.859	0.918	0.798
ASTRAL95 select <2.2 Å & P-score>3	64 (97%)	0.863	0.920	0.803
ASTRAL95 select & BLOSUM50	75 (100%)	0.829	0.885	0.730
ASTRAL95 select & BLOSUM62	75 (100%)	0.831	0.888	0.735
ASTRAL95 select & BLOSUM80	75 (100%)	0.831	0.884	0.731

The number of families with statistically significant (p<0.01) bilinear correlation coefficients (ratio). The median, upper quartile and lower quartile of the bilinear multiple correlation coefficients of these families are shown.

To evaluate different models, we calculated the adequacies (*r* ^2^, see Methods) of linear fitting (PNI and RMSD) versus bilinear fitting, bilinear fitting versus paraboloid fitting and bilinear fitting versus cubic spline function fitting, respectively. In analysis based on the accurate alignment results, the median adequacy for linear (PNI and RMSD) versus bilinear models is 0.885 (Table 2). The upper and lower quartiles are 0.932 and 0.808, respectively. For more than half of all the families, the bilinear model shows an improvement of more than 11% comparing to the linear model. For lower quartile families the improvement is near 20%. We also used paraboloid (constructed through introducing square terms in the bilinear model) and cubic spline functions to fit the data. The median of the adequacy for bilinear fitting versus paraboloid fit is 0.982. The median of the adequacy for bilinear fitting versus cubic spline function fitting is 0.988. The above analysis suggests that the bilinear model is superior to the linear model, while high-order fitting has no significant advantage compared with the bilinear model. A similar result was obtained when we analyzed all the alignment results (Table 2). The bilinear model accounts well for the combined effects of both substitutions and indels on structural changes. Moreover, this model has a significant improvement compared with the linear model which considers only substitutions.

**Table 2:** Adequacy analysis.

Adequacy		Median	Upper quartile	Lower quartile	r^2^>0.9%
ASTRAL95 select & P-score>3	Linear fit versus Bilinear fit	0.885	0.932	0.808	45.2
	Bilinear fit versus Paraboloid fit	0.982	0.995	0.960	97.3
	Bilinear fit versus Cubic spline function fit	0.998	1.114	0.948	87.7
ASTRAL95 select	Linear fit versus Bilinear fit	0.841	0.907	0.741	29.3
	Bilinear fit versus Paraboloid fit	0.973	0.988	0.951	98.7

The median, upper and lower quartiles of the adequacies of linear fitting versus bilinear fitting, bilinear fitting versus paraboloid fitting and bilinear fitting versus cubic spline function fitting are given. The right hand column shows the percentage of the families whose adequacies are above 0.9 (33/73, 71/73, 63/73, 22/75, 74/75, respectively).

Because both substitutions and indels among homologous proteins increase with divergence time, the partial linear relation between the two variables (PNI and SNG) may also have an influence on the bilinear correlation coefficient. Through analysis of the accurate alignment results it is revealed that only 3 families have a variance inflation factor (VIF) exceeding 4 (PAP, 4.1; BGC, 4.6; GAP, 5.0). Also, the PNI-RMSD partial correlation coefficients and SNG-RMSD partial correlation coefficients are significantly different from zero (p<0.01) in these families. So in our study, the influence arising from the co-linearity of the variables is not significant (Table S2).

Although for most of the families studied, the bilinear correlation of PNI and SNG versus RMSD is statistically significant, the bilinear multiple correlation coefficients are still highly varied among the families. The distribution of data within a family may have a significant influence on the bilinear correlation coefficients, and a low bilinear correlation coefficient may be produced if the range of PNI is very small. The two families which failed to yield statistically significant bilinear correlation coefficients have the smallest range of PNI (CBS: PNI standard deviation is 4.7; TCR: PNI standard deviation is 5.8). And the PNI standard deviations of 15 families whose bilinear correlation coefficients are below 0.75 are all lower than the median of all 75 families (14.2). In addition, there are no strong correlations between structure size (the average length of pairwise alignment within a family) and bilinear correlation (r = 0.42, p<0.01). Moreover, we have observed no correlation between either the number of gaps per unit length and bilinear correlation (r = −0.143, p = 0.23) or the total number of indels and bilinear correlation (r = 0.251, p = 0.03). We also investigated the possibility that if the stability of proteins is determined by strong interactions (such as disulfide bonds), the correlations between sequence and structure are likely to be weak [14]. However, in our study, the difference between the bilinear correlation coefficients of the families with 1.5 or more disulfide bonds (Sample 1, 13 families) and those with less than 1.5 disulfide bonds (Sample 2, 60 families) is not significant (*t*-test gives p = 0.34), hence disulfide bond does not have a significant influence on the bilinear correlation coefficients.

**Figure 4:**
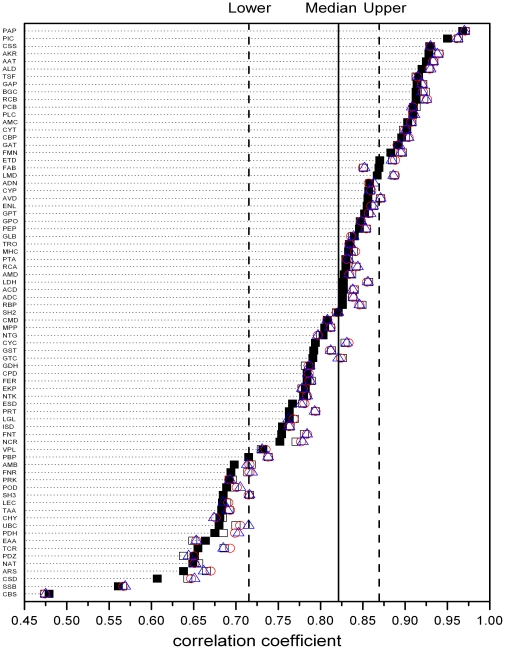
The sequence-structure correlation changes slightly under different substitution similarity assessing method. For all the 75 families, the bilinear multiple correlation coefficients produced by using PNI (▪), PNS-BLOSUM50 (□), PNS-BLOSUM62 (○) and PNS-BLOSUM80 (△) matrix differ slightly with each other. The solid line stands for the medians of the bilinear multiple correlation coefficients of PNI, SNG and RNSD, and the left and right dashed lines stand for the upper and down quartiles respectively.

In order to investigate the effects of amino acid similarity on the bilinear regression analysis, we used the similarity matrix to score the sequence alignments based on structure, and defined percent sequence non-similarity (PNS) in a similar way with PNI. The non-conservative substitutions are defined as the number and fraction of residues for which the alignment scores have zero or negative values. In all the 75 families, the bilinear multiple correlation coefficients and their medians are significant (p<0.01) using different similarity matrix (BLOSUM50, BLOSUM62 or BLOSUM80) [30] (Table 1). If the effect on structures from non-conservative substitutions is much more significant than that from conservative substitutions, the bilinear multiple correlation coefficients will increase by replacing PNI with PNS. However, in most of the families, there is no significant difference between bilinear multiple correlation coefficients produced by using PNI and PNS (different substitutions are measured by BLOSUM50, BLOSUM62 and BLOSUM80 matrix) (Figure 4). This result further supported the bilinear model, which means that for most families, relative to indels' effect on structure, the effects on structure from conservative substitutions per unit and non-conservative substitutions per unit are not significantly different.

In addition, we also used Z-score instead of RMSD to quantify structure variations. We analyzed the “PNI-SNG-Z-score” bilinear multiple correlation coefficients (Figure S1 and Table S3). In 66 of the 75 families we studied, the difference between “PNI-SNG-Z-score” bilinear multiple correlation coefficients and “PNI-SNG-RMSD” bilinear multiple correlation coefficients is less than 0.1. What's more, in 41 families, the R values of “PNI-SNG-Z-score” are even higher. Therefore, the bilinear correlation between sequence and structure is not due to our using RMSD to quantify the structure variations --- we can obtain similar result by using Z-score.

### Structural mutation sensitivity

For most families the bilinear model could well account for the combined effects of substitutions and indels on structures. The regression of PNI and SNG versus RMSD yields two coefficients, which could be used to evaluate the average structure influence per substitution and per indel in a sequence of standard length within a certain family. Thus we name the regression coefficients b_1_ and b_2_ as “structural substitution sensitivity” (SSS) and “structural indels sensitivity” (SIDS), respectively. These two names come from early work by Wood and Pearson. They defined the amount of structural change per unit sequence change as the structural mutation sensitivity [12]. They further discovered that structural mutation sensitivity varies 3.9-fold among different protein families. Moreover, the difference is not significantly correlated with protein structure class, the average protein size or the mutation rate in a protein family [12]. Through multiple comparison analysis, Panchenko et al pointed out that the regression coefficients do not show a statistical difference among protein families with a significant linear correlation [14].

**Figure 5:**
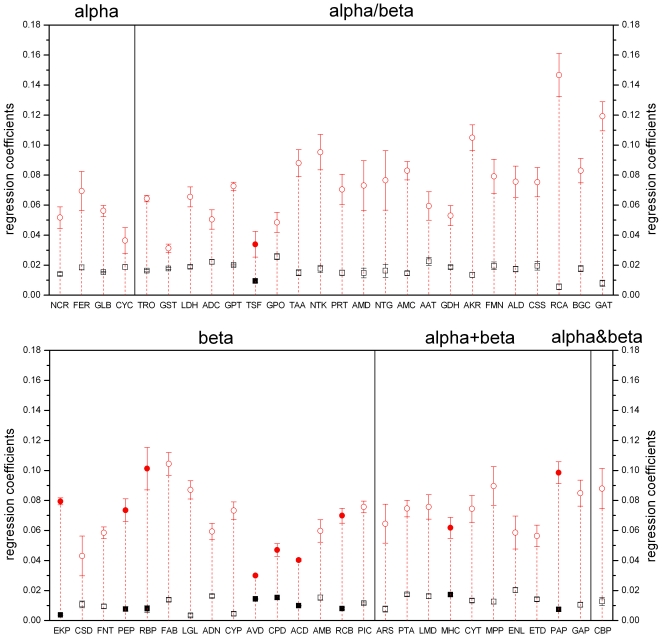
Neither SSS nor SIDS of 52 families is dependent on structural class. The figure shows SSS and SIDS (bilinear fit regression coefficients b_1_ and b_2_) and the standard deviation of these regression coefficients. Squares and circles represent SSS and SIDS respectively. Filled squares or circles indicate families where the average number of disulfide bonds is 1.5 or more; open squares or circles represent families with less than 1.5 disulfide bonds on average. Five regions corresponds to 5 SCOP structure classes. Family names are abbreviated as in Table S1.

To compare the regression coefficients of different families, we selected and studied 52 families with high bilinear correlation (R>0.75) and with statistically significant regression coefficients (p<0.01) (indicated by “†” in Table S1). Figure 5 shows the SSS and SIDS distribution in different structural classes in those families. Both SSS and SIDS show obvious differences among different families, while within the same family, the structural influence from each unit of indels is always larger than that from each unit of substitutions. The size of the structures show no correlation with SSS (r = −0.07, p = 0.60) and no strong correlation with SIDS (r = 0.43, p<0.01). The increase in frequency of indels per unit sequence shows no effect on SSS (r = −0.03, p = 0.85) or SIDS (r = 0.31, p = 0.03).

We conducted a *t*-test to examine the influence of strong interactions (e.g. disulfide bonds) on SSS and SIDS. Sample 1 consists of families with 1.5 or more disulfide bonds (10 families, indicated by filled squares or circles in Figure 5) and sample 2 is composed of families with less than 1.5 disulfide bonds (42 families). The result show that the average number of disulfide bonds in a family has a significant influence on SSS (p<0.01) but almost no influence on SIDS (p = 0.25). This phenomenon may arise because disulfide bonds usually result in stabilization of the hydrophobic core of protein. Since indels almost exclusively occur on the surface of proteins [14], [31], their effect on structures, i.e. SIDS, is not affected by the existence of disulfide bonds.

In those families (42 families) which are under nearly no restriction by disulfide bonds, there is a weak negative correlation between SSS and SIDS among different families (r = −0.46, p<0.01). One possible explanation for this restriction relationship between SSS and SIDS is that effects on structure produced by substitution of sequences near gaps can be covered by indels. The larger the effect indels per unit have on structures, the smaller the visible effects substitutions per unit would have on structures.

## Discussion

Wood and Pearson suggested that the discrepancy between the linear correlation observed in quantitative analyses based on statistical significance, and the non-linear relationship of RMSD versus percentage sequence identity may arise from three technical problems [12]: (1) it is hard to exclude structure changes caused by non-sequence factors, therefore even two identical sequences could have structure differences; (2) problems in protein structure alignments, which prevent us from always obtaining the best alignment; (3) sequence alignment algorithms cannot generate reliable alignments for remote sequences.

Considering problem (1), non-sequence factors are largely the atom position differences arising from the process of structure determination, including methods, position errors, and crystallization condition (e.g. ion strength, substrate binding condition). The atom position errors of low-resolution structure data from NMR and X-ray crystallization can be as high as 1 Å, while high-resolution X-ray crystallography can provide structures with errors <0.3 Å [32]. In order to reduce this effect, we chose structure data from the ASTRAL95 non-redundant database, in which every pair of structures has a sequence identity lower than 95%. The use of non-redundant data sets minimizes alignments between structures of high sequence identity, hence weakening the influence of non-sequence factors. Meanwhile, we selected all the high-resolution structures (better than 2.2 Å) from the whole database to study separately as a control, in order to reduce uncertainties from low resolution structures. In regard to problem (2), we chose the advanced structure matching program SSM, which is considered one of the best current structural alignment software packages [33], [34], [35]. We mainly selected those alignments with a P-score exceeding 3, as this is usually considered accurate [20]. In order to minimize problem (3), we employed a sequence alignment based on structure rather than a sequence alignment based on sequence. This is because the gaps generated in sequence-based alignment algorithms often do not represent real indels, especially for remote sequences. Since structures are more conserved than sequences, remote proteins are better matched by sequence alignment based on structure.

After minimizing the influence from these technique problems, if we perform regression analysis of PNI versus RMSD, a result similar to previous studies can still be obtained (Text S1) [7]. As a result, in addition to these technical problems causing nonlinear relationships between sequences and structures, we propose that there exists a more fundamental reason: besides PNI, the number of indels is another independent variable that contributes to structure variations in evolution. When studying how sequence variation influences structures, we should consider the combined influence from both amino acid substitutions and indels (Figure 2). We use a bilinear model to describe the influence of sequence variations on structural changes, and this provides better results in a wide range of proteins (75 structure families) (Table 1, Figure 3). Moreover, it provides a significant improvement compared with the linear model which takes into account only substitutions (Table 2).

In the evolutionary process, the substitution rate of biological molecules (amino acids and nucleotides) is considered stable, which is called the “molecular clock” hypothesis. We showed that the correlation of sequence variation and structure changes could be described by a bilinear model. This suggest that the rate of protein structure variations can also be considered stable. In sum, these results can deepen our understanding to the protein structure evolutionary mechanisms.

## Methods

### Selection of structure families

The homologous protein families studied were taken from SCOP (version 1.73), the Structural Classification Of Proteins database [21], [22]. Structural data come from ASTRAL non-redundant database [23], with less than 95% identity to each of its sequences. From the complete set of 3464 SCOP families we first selected all the 121 families which each contain 20 or more non-redundant structures according to the ASTRAL95 non-redundant database. Then we queried all the related information on these families to remove the mutated structures and structures determined by NMR. This left 76 families including 20 or more non-redundant structures. We submitted these families' structures to the Secondary Structure Matching tool (SSM) online comparison service to do pairwise alignment within family. Family a.121.1.1 had less than 10 alignments with P-score>3 and was excluded. Sequence-structure correlation analysis was performed on the remaining group of 75 families which we called “ASTRAL95 select”. Family b.1.1.1 is an exception because there are 523 non-redundant structures in the family, which would yield too many pairwise alignments to analyze. Thus only the 213 high-resolution structures were selected for analysis. These 75 families contain 3179 structures in total, accounting for 20.8% of the ASTRAL95 non-redundant structure database.

Further refinement gave us a second test set. We examined high-resolution structures from the “ASTRAL95 select” set; i.e. those with crystal resolution better than 2.2 Å. Nine families were excluded because the number of accurate alignments with P-score greater than 3 was below 10. The remaining 66 families were called “ASTRAL95 select <2.2 Å”.

### Structure comparisons

Pairwise alignment of selected structures in homologous protein families was also conducted through SSM online services. We selected and analyzed only the “accurate alignments”: those with P-score greater than 3. The P-score is the negative logarithm of the *p*-value, which accounts for RMSD, number of aligned residues (*N*_algn_) and number of gaps in the alignment (*N*_gap_).

RMSD is obtained from the alignments result. The percentage of non-identity (PNI) and the standardized number of gaps (SNG) were calculated as follows:
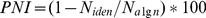
(1)

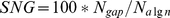
(2)


where *N*_gap_ is total number of gaps in alignments irrespective of the length of each individual gap, *N*_algn_ is the number of amino acid residues in the alignment, and *N*_iden_ is the number of identical sites in the alignments. We compiled a PERL program to extract the total gap number.

Information on disulfide bonds comes from Protein Data Bank files of the selected proteins. The numbers of disulfide bonds in each protein is defined as the number of disulfide bonds in the local structural domain. This data was extracted through a PERL program compiled by ourselves. ‘Average number of disulfide bonds’ in each family is the average number of disulfide bonds in alignment sequences with P-score greater than 3 in all the pairwise alignments.

All related data mentioned above could be obtained from http://202.194.15.140/research/.

### Regression analysis

Statistical analysis was conducted using SigmaStat 3.5. To study the combined effects of substitutions and indels on structures we further performed correlation and regression analysis. The bilinear correlation coefficients, PNI-RMSD partial correlation coefficients and SNG-RMSD partial correlation coefficients were calculated. The *p*-values were calculated under the null hypothesis that the correlation coefficients equals zero.

Adequacy (*r* ^2^) analysis was used to quantify the improvements from using the bilinear model when compared with the linear model which considered only substitution. We also studied the results of using high order fits, compared to the bilinear model. High order fitting of the data was performed both via a paraboloid equation (constructed through introducing a squared term in the bilinear model) or the cubic spline function. Adequacies of linear fitting versus bilinear fitting, bilinear fitting versus paraboloid fitting and bilinear fitting versus cubic spline function fitting were calculated as follows:
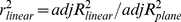
(3)

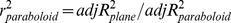
(4)

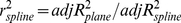
(5)


Where 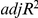
is the coefficient of determination adjusted according to the number of data points and the number of independent variables in the linear, bilinear, paraboloid or cubic spline function fitting. Each coefficient of determination (*R* ^2^) was adjusted by:
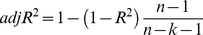
(6)


where n is the number of data points and k is the number of independent variables in the fitting.

## Supporting Information

Table S1 Selected protein families with associated statistical parameters.
(0.13 MB DOC)

Table S2 The detection of co-linearity.
(0.13 MB DOC)

Table S3 Bilinear correlation coefficients of PNI-SNG-Z-score and bilinear correlation coefficients of PNI-SNG-RMSD.
(0.03 MB DOC)

Figure S1 Bilinear correlation between PNI-SNG-Z-score. We didn't obtain a significantly lower bilinear correlation coefficient in the whole alignment results of all the 75 families, when the Z-score is used to characterize the structure changes instead of RMSD.
(2.75 MB DOC)

Text S1 After trying to weaken the influence of these technique problems, if making the regression analysis on PNI and RMSD merely according to our data, a result similar to the former researchers can still be obtained.
(0.16 MB DOC)
