# Supplementary material for: The Combined Effects of Amino Acid Substitutions and Indels on the Evolution of Structure within Protein Families
Source: PLoS One. 2010 Dec 13;5(12):e14316. doi: 10.1371/journal.pone.0014316 (PMC3001449; doi:10.1371/journal.pone.0014316)
Supplement: Table S1 — Selected protein families with associated statistical parameters. (0.13 MB DOC) [file pone.0014316.s001.doc]

**Table S1.** **Selected protein families with associated statistical parameters.**

| **SCOP^a^** | **Code** | **Family names** | **PDB^b^** | **N^c^** | **Corr^d^** | **b_1_^e^** | **b_2_^f^** |
| --- | --- | --- | --- | --- | --- | --- | --- |
| a.1.1.2^†^ | GLB | Globins | 73 | 1352 | 0.88 | 0.0153** | 0.0562** |
| a.1.1.3 | PCB | Phycocyanin-like phycobilisome proteins | 23 | 166 | 0.89 | 0.0208** | 0.0085 |
| a.3.1.1^†^ | CYC | monodomain cytochrome c | 36 | 136 | 0.90 | 0.0187** | 0.0364** |
| a.4.1.9 | TCR | Tetracyclin repressor-like, N-terminal domain | 26 | 47 | 0.38 |  |  |
| a.25.1.1^†^ | FER | Ferritin | 31 | 189 | 0.83 | 0.0185** | 0.0694** |
| a.39.1.5 | CMD | Calmodulin-like | 31 | 36 | 0.93 | 0.0015 | 0.1868** |
| a.45.1.1 | GTC | Glutathione S-transferase (GST), C-terminal domain | 50 | 242 | 0.86 | 0.0198** | 0.0107 |
| a.123.1.1^†^ | NCR | Nuclear receptor ligand-binding domain | 33 | 359 | 0.79 | 0.0141** | 0.0517** |
| a.133.1.2 | VPL | Vertebrate phospholipase A2 | 43 | 819 | 0.67 | 0.0092** | 0.0213** |
| b.1.1.1^†^ | AVD | V set domains (antibody variable domain-like) | 523 | 20814 | 0.86 | 0.0144** | 0.0300** |
| b.1.1.2^†^ | ACD | C1 set domains (antibody constant domain-like) | 130 | 5754 | 0.88 | 0.0099** | 0.0403** |
| b.1.1.4 | ISD | I set domains | 58 | 424 | 0.67 | 0.0137** | 0.0365** |
| b.1.2.1^†^ | FNT | Fibronectin type III | 39 | 309 | 0.80 | 0.0094** | 0.0585** |
| b.1.18.2 | ESD | E-set domains of sugar-utilizing enzymes | 24 | 18 | 0.93 | 0.0159** | 0.0498 |
| b.6.1.1 | PLC | Plastocyanin/azurin-like | 25 | 124 | 0.89 | 0.0039* | 0.0716** |
| b.6.1.3^†^ | CPD | Multidomain cupredoxins | 44 | 436 | 0.88 | 0.0153** | 0.0470** |
| b.29.1.1^†^ | LGL | Legume lectins | 23 | 192 | 0.85 | 0.0034** | 0.0871** |
| b.34.2.1 | SH3 | SH3-domain | 36 | 430 | 0.64 | 0.0079** | 0.0421** |
| b.35.1.2^†^ | ADN | Alcohol dehydrogenase-like, N-terminal domain | 29 | 358 | 0.85 | 0.0163** | 0.0593** |
| b.36.1.1 | PDZ | PDZ domain | 23 | 253 | 0.65 | 0.0085** | 0.0671** |
| b.40.4.3 | SSB | Single strand DNA-binding domain, SSB | 27 | 20 | 0.76 | 0.0104* | 0.0190 |
| b.40.4.5^†^ | CSD | Cold shock DNA-binding domain-like | 29 | 54 | 0.79 | 0.0107** | 0.0430** |
| b.42.2.1^†^ | RCB | Ricin B-like | 28 | 218 | 0.93 | 0.0080** | 0.0699** |
| b.45.1.1 | POD | PNP-oxidase like | 21 | 119 | 0.68 | 0.0245** | 0.0129 |
| b.47.1.2^†^ | EKP | Eukaryotic proteases | 49 | 1170 | 0.78 | 0.0038** | 0.0794** |
| b.50.1.2^†^ | PEP | Pepsin-like | 24 | 204 | 0.81 | 0.0077** | 0.0735** |
| b.60.1.1^†^ | RBP | Retinol binding protein-like | 24 | 91 | 0.82 | 0.0079** | 0.1012** |
| b.60.1.2^†^ | FAB | Fatty acid binding protein-like | 23 | 248 | 0.85 | 0.0138** | 0.1044** |
| b.62.1.1^†^ | CYP | Cyclophilin (peptidylprolyl isomerase) | 22 | 227 | 0.85 | 0.0045** | 0.0733** |
| b.71.1.1^†^ | AMB | Alpha-Amylases, C-terminal beta-sheet domain | 41 | 110 | 0.92 | 0.0153** | 0.0597** |
| b.121.4.1^†^ | PIC | Picornaviridae-like VP (VP1, VP2, VP3 and VP4) | 39 | 279 | 0.95 | 0.0116** | 0.0757** |
| c.1.4.1^†^ | FMN | FMN-linked oxidoreductases | 24 | 73 | 0.94 | 0.0196** | 0.0792** |
| c.1.7.1^†^ | AKR | Aldo-keto reductases (NADP) | 21 | 175 | 0.93 | 0.0136** | 0.1049** |
| c.1.8.1^†^ | AMC | Amylase, catalytic domain | 46 | 386 | 0.92 | 0.0146** | 0.0830** |
| c.1.8.3^†^ | BGC | beta-glycanases | 41 | 148 | 0.96 | 0.0177** | 0.0830** |
| c.1.10.1^†^ | ALD | Class I aldolase | 32 | 93 | 0.94 | 0.0174** | 0.0756** |
| c.2.1.1^†^ | ADC | Alcohol dehydrogenase-like, C-terminal domain | 29 | 353 | 0.84 | 0.0222** | 0.0505** |
| c.2.1.2^†^ | TRO | Tyrosine-dependent oxidoreductases | 98 | 3044 | 0.81 | 0.0163** | 0.0643** |
| c.2.1.3^†^ | GDH | Glyceraldehyde-3-phosphate dehydrogenase-like, N-terminal domain | 50 | 234 | 0.93 | 0.0187** | 0.0530** |
| c.2.1.5^†^ | LDH | LDH N-terminal domain-like | 30 | 413 | 0.82 | 0.0189** | 0.0655** |
| c.2.1.6 | PDH | 6-phosphogluconate dehydrogenase-like, N-terminal domain | 20 | 92 | 0.72 | 0.0292** | 0.0331* |
| c.2.1.7^†^ | AMD | Aminoacid dehydrogenase-like, C-terminal domain | 21 | 55 | 0.90 | 0.0148** | 0.0731** |
| c.3.1.5 | FNR | FAD/NAD-linked reductases, N-terminal and central domains | 47 | 493 | 0.74 | 0.0119** | 0.0527** |
| c.23.1.1 | CHY | CheY-related | 24 | 256 | 0.60 | 0.0191** | 0.0471** |
| c.37.1.1^†^ | NTK | Nucleotide and nucleoside kinases | 42 | 128 | 0.88 | 0.0177** | 0.0953** |
| c.37.1.8^†^ | GPT | G proteins | 99 | 2144 | 0.85 | 0.0201** | 0.0726** |
| c.37.1.10^†^ | NTG | Nitrogenase iron protein-like | 27 | 46 | 0.91 | 0.0163** | 0.0765** |
| c.37.1.11^†^ | RCA | RecA protein-like (ATPase-domain) | 25 | 61 | 0.95 | 0.0056** | 0.1467** |
| c.37.1.19^†^ | TAA | Tandem AAA-ATPase domain | 49 | 173 | 0.87 | 0.0149** | 0.0880** |
| c.37.1.20 | EAA | Extended AAA-ATPase domain | 29 | 26 | 0.80 | 0.0136** | 0.0775* |
| c.47.1.5^†^ | GST | Glutathione S-transferase (GST), N-terminal domain | 45 | 783 | 0.81 | 0.0178** | 0.0312** |
| c.47.1.10^†^ | GPO | Glutathione peroxidase-like | 31 | 244 | 0.86 | 0.0257** | 0.0485** |
| c.61.1.1^†^ | PRT | Phosphoribosyltransferases (PRTases) | 31 | 106 | 0.89 | 0.0149** | 0.0704** |
| c.67.1.1^†^ | AAT | AAT-like | 25 | 123 | 0.92 | 0.0225** | 0.0594** |
| c.67.1.3^†^ | CSS | Cystathionine synthase-like | 21 | 68 | 0.95 | 0.0196** | 0.0753** |
| c.67.1.4^†^ | GAT | GABA-aminotransferase-like | 22 | 50 | 0.97 | 0.0079** | 0.1192** |
| c.94.1.1 | PBP | Phosphate binding protein-like | 52 | 64 | 0.82 | 0.0073* | 0.1089** |
| c.94.1.2^†^ | TSF | Transferrin | 20 | 128 | 0.85 | 0.0095** | 0.0339** |
| d.3.1.1^†^ | PAP | Papain-like | 23 | 210 | 0.94 | 0.0075** | 0.0985** |
| d.19.1.1^†^ | MHC | MHC antigen-recognition domain | 64 | 692 | 0.89 | 0.0172** | 0.0618** |
| d.20.1.1 | UBC | UBC-related | 33 | 466 | 0.65 | 0.0160** | 0.0264** |
| d.32.1.3^†^ | ETD | Extradiol dioxygenases | 22 | 116 | 0.92 | 0.0141** | 0.0564** |
| d.37.1.1 | CBS | CBS-domain | 25 | 94 | 0.06 |  |  |
| d.54.1.1^†^ | ENL | Enolase N-terminal domain-like | 24 | 128 | 0.91 | 0.0203** | 0.0586** |
| d.81.1.1^†^ | GAP | GAPDH-like | 24 | 175 | 0.94 | 0.0105** | 0.0849** |
| d.93.1.1 | SH2 | SH2 domain | 26 | 217 | 0.71 | 0.0115** | 0.0406** |
| d.104.1.1^†^ | ARS | Class II aminoacyl-tRNA synthetase (aaRS)-like, catalytic domain | 28 | 96 | 0.80 | 0.0077** | 0.0645** |
| d.108.1.1 | NAT | N-acetyl transferase, NAT | 53 | 412 | 0.60 | 0.0185** | 0.0392** |
| d.144.1.7 | PRK | Protein kinases, catalytic subunit | 51 | 803 | 0.71 | 0.0127** | 0.0610** |
| d.153.1.4^†^ | PTA | Proteasome subunits | 35 | 541 | 0.80 | 0.0173** | 0.0746** |
| d.162.1.1^†^ | LMD | Lactate & malate dehydrogenases, C-terminal domain | 25 | 219 | 0.83 | 0.0162** | 0.0757** |
| d.165.1.1^†^ | CYT | Plant cytotoxins | 20 | 187 | 0.90 | 0.0133** | 0.0744** |
| d.169.1.1 | LEC | C-type lectin domain | 51 | 877 | 0.67 | 0.0084** | 0.0506** |
| d.185.1.1^†^ | MPP | MPP-like | 20 | 65 | 0.90 | 0.0126** | 0.0896** |
| e.3.1.1^†^ | CBP | beta-Lactamase/D-ala carboxypeptidase | 27 | 65 | 0.92 | 0.0127** | 0.0879** |

^a^ Classification number in SCOP 1.73

^b^ Number of selected structures within families

^c^ Number of accurate alignments within families

^d^ Bilinear correlation coefficient of a family

^e^ Regression factor b_1_ from bilinear regression (SSS)

^f^ Regression factor b_2_ from bilinear regression (SIDS)

In Table S1 we list the SCOP classification number of 75 protein families, SCOP family names, abbreviations of family names, number of selected structures, number of accurate alignments (for definition, see Methods - Structure Comparisons) and bilinear correlation coefficient, regression factors b_1_ and b_2_ from bilinear regression of PNI and SNG versus RMSD. Families a.4.1.9 and d.37.1.1 failed to yield statistically significant bilinear correlation coefficients. The significance of the partial correlation coefficients is the same as that of the regression coefficients. A *p*-value<0.05 is indicated by "*", and "**" indicates a *p*-value<0.01. Those families having high bilinear coefficients (R>0.75) and also high significance of both b_1_ and b_2_ regression coefficients (p<0.01) are marked by "†".
