# Supplementary material for: The Combined Effects of Amino Acid Substitutions and Indels on the Evolution of Structure within Protein Families
Source: PLoS One. 2010 Dec 13;5(12):e14316. doi: 10.1371/journal.pone.0014316 (PMC3001449; doi:10.1371/journal.pone.0014316)
Supplement: Table S2 — The detection of co-linearity. (0.13 MB DOC) [file pone.0014316.s002.doc]

**Table S2. The detection of co-linearity.**

| **SCOP** ^a^ | **Code** | **N** ^b^ | **R** ^c^ | ***p*** ^c^ | **VIF**^d^ | **b_1_** ^e^ | ***p*** ^e^ | **b_2_** ^f^ | ***p*** ^f^ |
| --- | --- | --- | --- | --- | --- | --- | --- | --- | --- |
| a.1.1.2 | GLB | 1352 | 0.881 | <0.001 | 1.960 | 0.0153 | <0.0001 | 0.0562 | <0.0001 |
| a.1.1.3 | PCB | 166 | 0.894 | <0.001 | 1.794 | 0.0208 | <0.0001 | 0.0085 | **0.5174** |
| a.3.1.1 | CYC | 136 | 0.899 | <0.001 | 2.509 | 0.0187 | <0.0001 | 0.0364 | <0.0001 |
| a.4.1.9 | TCR | 47 | 0.381 | **0.032** | 1.005 |  |  |  |  |
| a.25.1.1 | FER | 189 | 0.835 | <0.001 | 1.950 | 0.0185 | <0.0001 | 0.0694 | <0.0001 |
| a.39.1.5 | CMD | 36 | 0.927 | <0.001 | 1.390 | 0.0015 | **0.4608** | 0.1868 | <0.0001 |
| a.45.1.1 | GTC | 242 | 0.862 | <0.001 | 1.712 | 0.0198 | <0.0001 | 0.0107 | **0.2709** |
| a.123.1.1 | NCR | 359 | 0.786 | <0.001 | 1.462 | 0.0141 | <0.0001 | 0.0517 | <0.0001 |
| a.133.1.2 | VPL | 819 | 0.667 | <0.001 | 2.298 | 0.0092 | <0.0001 | 0.0213 | <0.0001 |
| b.1.1.1 | AVD | 20814 | 0.860 | <0.001 | 3.477 | 0.0144 | <0.0001 | 0.0300 | <0.0001 |
| b.1.1.2 | ACD | 5754 | 0.878 | <0.001 | 2.155 | 0.0099 | <0.0001 | 0.0403 | <0.0001 |
| b.1.1.4 | ISD | 424 | 0.673 | <0.001 | 1.250 | 0.0137 | <0.0001 | 0.0365 | <0.0001 |
| b.1.2.1 | FNT | 309 | 0.802 | <0.001 | 1.418 | 0.0094 | <0.0001 | 0.0585 | <0.0001 |
| b.1.18.2 | ESD | 18 | 0.927 | <0.001 | 3.038 | 0.0159 | 0.0023 | 0.0498 | **0.054** |
| b.6.1.1 | PLC | 124 | 0.891 | <0.001 | 3.379 | 0.0039 | **0.0377** | 0.0716 | <0.0001 |
| b.6.1.3 | CPD | 436 | 0.878 | <0.001 | 2.271 | 0.0153 | <0.0001 | 0.0470 | <0.0001 |
| b.29.1.1 | LGL | 192 | 0.852 | <0.001 | 1.803 | 0.0034 | 0.0009 | 0.0871 | <0.0001 |
| b.34.2.1 | SH3 | 430 | 0.642 | <0.001 | 1.204 | 0.0079 | <0.0001 | 0.0421 | <0.0001 |
| b.35.1.2 | ADN | 358 | 0.853 | <0.001 | 1.855 | 0.0163 | <0.0001 | 0.0593 | <0.0001 |
| b.36.1.1 | PDZ | 253 | 0.651 | <0.001 | 1.311 | 0.0085 | <0.0001 | 0.0671 | <0.0001 |
| b.40.4.3 | SSB | 20 | 0.761 | <0.001 | 3.185 | 0.0104 | **0.0385** | 0.0190 | **0.5962** |
| b.40.4.5 | CSD | 54 | 0.793 | <0.001 | 1.703 | 0.0107 | <0.0001 | 0.0430 | 0.0021 |
| b.42.2.1 | RCB | 218 | 0.934 | <0.001 | 2.813 | 0.0080 | <0.0001 | 0.0699 | <0.0001 |
| b.45.1.1 | POD | 119 | 0.680 | <0.001 | 1.453 | 0.0245 | <0.0001 | 0.0129 | **0.2858** |
| b.47.1.2 | EKP | 1170 | 0.782 | <0.001 | 1.288 | 0.0038 | <0.0001 | 0.0794 | <0.0001 |
| b.50.1.2 | PEP | 204 | 0.809 | <0.001 | 1.934 | 0.0077 | <0.0001 | 0.0735 | <0.0001 |
| b.60.1.1 | RBP | 91 | 0.821 | <0.001 | 1.869 | 0.0079 | 0.0008 | 0.1012 | <0.0001 |
| b.60.1.2 | FAB | 248 | 0.847 | <0.001 | 1.297 | 0.0138 | <0.0001 | 0.1044 | <0.0001 |
| b.62.1.1 | CYP | 227 | 0.854 | <0.001 | 2.473 | 0.0045 | 0.0004 | 0.0733 | <0.0001 |
| b.71.1.1 | AMB | 110 | 0.924 | <0.001 | 2.413 | 0.0153 | <0.0001 | 0.0597 | <0.0001 |
| b.121.4.1 | PIC | 279 | 0.954 | <0.001 | 2.479 | 0.0116 | <0.0001 | 0.0757 | <0.0001 |
| c.1.4.1 | FMN | 73 | 0.941 | <0.001 | 2.828 | 0.0196 | <0.0001 | 0.0792 | <0.0001 |
| c.1.7.1 | AKR | 175 | 0.930 | <0.001 | 2.950 | 0.0136 | <0.0001 | 0.1049 | <0.0001 |
| c.1.8.1 | AMC | 386 | 0.917 | <0.001 | 3.826 | 0.0146 | <0.0001 | 0.0830 | <0.0001 |
| c.1.8.3 | BGC | 148 | 0.958 | <0.001 | **4.599** | 0.0177 | <0.0001 | 0.0830 | <0.0001 |
| c.1.10.1 | ALD | 93 | 0.943 | <0.001 | 2.705 | 0.0174 | <0.0001 | 0.0756 | <0.0001 |
| c.2.1.1 | ADC | 353 | 0.838 | <0.001 | 1.907 | 0.0222 | <0.0001 | 0.0505 | <0.0001 |
| c.2.1.2 | TRO | 3044 | 0.807 | <0.001 | 2.165 | 0.0163 | <0.0001 | 0.0643 | <0.0001 |
| c.2.1.3 | GDH | 234 | 0.930 | <0.001 | 2.980 | 0.0187 | <0.0001 | 0.0530 | <0.0001 |
| c.2.1.5 | LDH | 413 | 0.823 | <0.001 | 1.588 | 0.0189 | <0.0001 | 0.0655 | <0.0001 |
| c.2.1.6 | PDH | 92 | 0.718 | <0.001 | 1.643 | 0.0292 | <0.0001 | 0.0331 | **0.0146** |
| c.2.1.7 | AMD | 55 | 0.904 | <0.001 | 3.140 | 0.0148 | <0.0001 | 0.0731 | <0.0001 |
| c.3.1.5 | FNR | 493 | 0.738 | <0.001 | 1.546 | 0.0119 | <0.0001 | 0.0527 | <0.0001 |
| c.23.1.1 | CHY | 256 | 0.599 | <0.001 | 1.249 | 0.0191 | <0.0001 | 0.0471 | <0.0001 |
| c.37.1.1 | NTK | 128 | 0.883 | <0.001 | 2.057 | 0.0177 | <0.0001 | 0.0953 | <0.0001 |
| c.37.1.8 | GPT | 2144 | 0.845 | <0.001 | 1.708 | 0.0201 | <0.0001 | 0.0726 | <0.0001 |
| c.37.1.10 | NTG | 46 | 0.911 | <0.001 | 3.872 | 0.0163 | 0.0005 | 0.0765 | 0.0004 |
| c.37.1.11 | RCA | 61 | 0.947 | <0.001 | 3.138 | 0.0056 | 0.0045 | 0.1467 | <0.0001 |
| c.37.1.19 | TAA | 173 | 0.866 | <0.001 | 1.972 | 0.0149 | <0.0001 | 0.0880 | <0.0001 |
| c.37.1.20 | EAA | 26 | 0.797 | <0.001 | 1.834 | 0.0136 | 0.0062 | 0.0775 | **0.0493** |
| c.47.1.5 | GST | 783 | 0.811 | <0.001 | 1.573 | 0.0178 | <0.0001 | 0.0312 | <0.0001 |
| c.47.1.10 | GPO | 244 | 0.859 | <0.001 | 1.899 | 0.0257 | <0.0001 | 0.0485 | <0.0001 |
| c.61.1.1 | PRT | 106 | 0.894 | <0.001 | 2.055 | 0.0149 | <0.0001 | 0.0704 | <0.0001 |
| c.67.1.1 | AAT | 123 | 0.919 | <0.001 | 3.132 | 0.0225 | <0.0001 | 0.0594 | <0.0001 |
| c.67.1.3 | CSS | 68 | 0.945 | <0.001 | 2.860 | 0.0196 | <0.0001 | 0.0753 | <0.0001 |
| c.67.1.4 | GAT | 50 | 0.967 | <0.001 | 2.800 | 0.0079 | 0.0004 | 0.1192 | <0.0001 |
| c.94.1.1 | PBP | 64 | 0.819 | <0.001 | 1.869 | 0.0073 | **0.0177** | 0.1089 | <0.0001 |
| c.94.1.2 | TSF | 128 | 0.847 | <0.001 | 2.403 | 0.0095 | <0.0001 | 0.0339 | 0.0001 |
| d.3.1.1 | PAP | 210 | 0.938 | <0.001 | **4.066** | 0.0075 | <0.0001 | 0.0985 | <0.0001 |
| d.19.1.1 | MHC | 692 | 0.894 | <0.001 | 3.872 | 0.0172 | <0.0001 | 0.0618 | <0.0001 |
| d.20.1.1 | UBC | 466 | 0.648 | <0.001 | 1.782 | 0.0160 | <0.0001 | 0.0264 | 0.0002 |
| d.32.1.3 | ETD | 116 | 0.919 | <0.001 | 1.910 | 0.0141 | <0.0001 | 0.0564 | <0.0001 |
| d.37.1.1 | CBS | 94 | 0.063 | **0.837** | 1.022 |  |  |  |  |
| d.54.1.1 | ENL | 128 | 0.909 | <0.001 | 1.988 | 0.0203 | <0.0001 | 0.0586 | <0.0001 |
| d.81.1.1 | GAP | 175 | 0.940 | <0.001 | **5.004** | 0.0105 | <0.0001 | 0.0849 | <0.0001 |
| d.93.1.1 | SH2 | 217 | 0.713 | <0.001 | 1.272 | 0.0115 | <0.0001 | 0.0406 | <0.0001 |
| d.104.1.1 | ARS | 96 | 0.799 | <0.001 | 2.416 | 0.0077 | 0.0003 | 0.0645 | <0.0001 |
| d.108.1.1 | NAT | 412 | 0.601 | <0.001 | 1.248 | 0.0185 | <0.0001 | 0.0392 | <0.0001 |
| d.144.1.7 | PRK | 803 | 0.707 | <0.001 | 1.623 | 0.0127 | <0.0001 | 0.0610 | <0.0001 |
| d.153.1.4 | PTA | 541 | 0.803 | <0.001 | 1.405 | 0.0173 | <0.0001 | 0.0746 | <0.0001 |
| d.162.1.1 | LMD | 219 | 0.825 | <0.001 | 1.476 | 0.0162 | <0.0001 | 0.0757 | <0.0001 |
| d.165.1.1 | CYT | 187 | 0.900 | <0.001 | 2.591 | 0.0133 | <0.0001 | 0.0744 | <0.0001 |
| d.169.1.1 | LEC | 877 | 0.670 | <0.001 | 1.293 | 0.0084 | <0.0001 | 0.0506 | <0.0001 |
| d.185.1.1 | MPP | 65 | 0.900 | <0.001 | 1.616 | 0.0126 | <0.0001 | 0.0896 | <0.0001 |
| e.3.1.1 | CBP | 65 | 0.922 | <0.001 | 3.101 | 0.0127 | <0.0001 | 0.0879 | <0.0001 |

^a^ Classification number in SCOP 1.73

^b^ Number of accurate alignments within families

^c^ Bilinear correlation coefficient of a family and its statistically significance

^d^ VIF — variance inflation factors

^e^ Regression factor b_1_ from bilinear regression (SSS) and its statistically significance

^f^ Regression factor b_2_ from bilinear regression (SIDS) and its statistically significance
