# Supplementary material for: The Combined Effects of Amino Acid Substitutions and Indels on the Evolution of Structure within Protein Families
Source: PLoS One. 2010 Dec 13;5(12):e14316. doi: 10.1371/journal.pone.0014316 (PMC3001449; doi:10.1371/journal.pone.0014316)
Supplement: Table S3 — Bilinear correlation coefficients of PNI-SNG-Z-score and bilinear correlation coefficients of PNI-SNG-RMSD. (0.03 MB DOC) [file pone.0014316.s003.doc]

**Table S3. Bilinear correlation coefficients of PNI-SNG-Z-score and bilinear correlation coefficients of PNI-SNG-RMSD.**

|  | Median | Upper quartile | Lower quartile |
| --- | --- | --- | --- |
| PNI-SNG-Z-score | 0.817 | 0.878 | 0.723 |
| PNI-SNG-RMSD | 0.821 | 0.869 | 0.715 |
