# Supplementary material for: The Combined Effects of Amino Acid Substitutions and Indels on the Evolution of Structure within Protein Families
Source: PLoS One. 2010 Dec 13;5(12):e14316. doi: 10.1371/journal.pone.0014316 (PMC3001449; doi:10.1371/journal.pone.0014316)
Supplement: Figure S1 — Bilinear correlation between PNI-SNG-Z-score. We didn't obtain a significantly lower bilinear correlation coefficient in the whole alignment results of all the 75 families, when the Z-score is used to characterize the structure changes instead of RMSD. (2.75 MB DOC) [file pone.0014316.s004.doc]

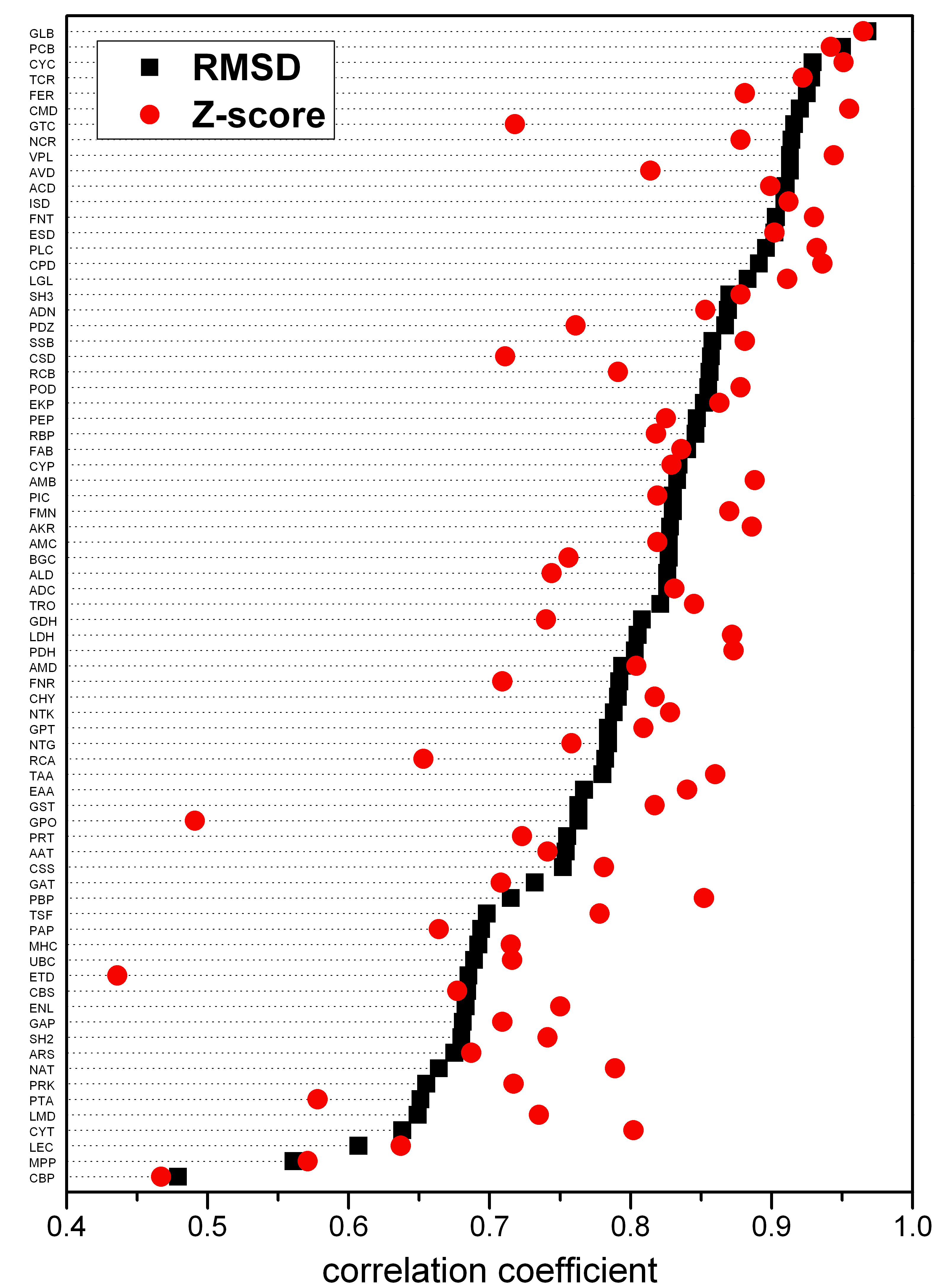


**Figure S1. Bilinear correlation between PNI-SNG-Z-score.** We didn’t obtain a significantly lower bilinear correlation coefficient in the whole alignment results of all the 75 families, when the Z-score is used to characterize the structure changes instead of RMSD.
