# Supplementary material for: The Combined Effects of Amino Acid Substitutions and Indels on the Evolution of Structure within Protein Families
Source: PLoS One. 2010 Dec 13;5(12):e14316. doi: 10.1371/journal.pone.0014316 (PMC3001449; doi:10.1371/journal.pone.0014316)
Supplement: Text S1 — After trying to weaken the influence of these technique problems, if making the regression analysis on PNI and RMSD merely according to our data, a result similar to the former researchers can still be obtained. (0.16 MB DOC) [file pone.0014316.s005.doc]

If only doing the regression analysis of PNI and RMSD, we obtained the data very similar to the former researches. In Figure S2, the correlation of PNI and RMSD of all the 50359 accurate alignment are shown. The following exponential relation were produced by least square fitting of the data:

RMSD=0.51exp(0.0161PNI)

**(7)**

In this equation the unit of RMSD is Å, PNI is expressed by percentage. There is an average 0.2Å difference between the RMSD value inferred by equation (8) and the observed RMSD value. The equation (7) can also be expressed as:

RMSD=0.51exp(1.61H)

**(8)**

In this equation, H represents mutation rate, that is H=PNI/100. This relationship is similar with the alignment results based on 36 alignment by Chothia and Lesk:

RMSD=0.4exp(1.87H)

**(9)**

There is an average 0.7Å difference between the RMSD value inferred by equation (9) and the observed RMSD value.


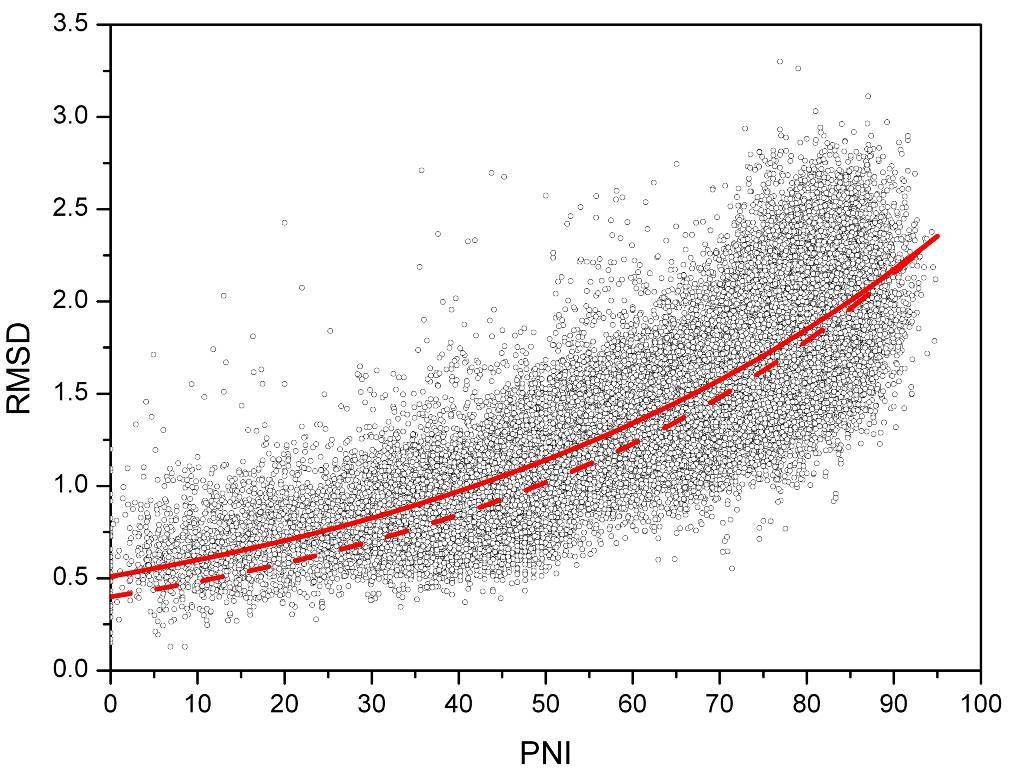


**Figure S2**. **The correlation between PNI and RMSD of all the 73 families expressed by 50359 reasonable alignment results.** The real line expresses the stimulated correlation by least squares method: RMSD=0.51exp(0.0161PNI). The dotted line represents the equation deduced by Chothia and Lesk.
